# Identification of and solution for false D‐dimer results

**DOI:** 10.1002/jcla.23216

**Published:** 2020-01-22

**Authors:** Xian‐Yan Zhang, Xue‐Xuan Zhang, Jia‐Long Xu, Teng‐Yi Huang, Ying Wu, Ye‐Ru Yang, Huan‐Bin Zhou, Ying‐E Wu

**Affiliations:** ^1^ Department of Laboratory Medicine The First Affiliated Hospital of Shantou University Medical College Shantou China; ^2^ Laboratory of Molecular Cardiology The First Affiliated Hospital of Shantou University Medical College Shantou China; ^3^ Laboratory of Molecular Imaging the First Affiliated Hospital of Shantou University Medical College Shantou China; ^4^ Department of Laboratory Medicine The Second Affiliated Hospital of Shantou University Medical College Shantou China

**Keywords:** 95% CI, D‐dimer, false result, fibrin degradation product, heterophilic antibody

## Abstract

**Background:**

Clinically, D‐dimer (DD) levels are mainly used to exclude diseases such as deep venous thrombosis (DVT). In clinical testing, DD assays can be subjected to interference that may cause false results, which directly affect the clinical diagnosis. Our hypothesis was that the 95% confidence intervals (CIs) of the fibrin degradation product (FDP)/DD and fibrinogen (Fib)/DD ratios were used to identify these false results and corrected via multiple dilutions.

**Methods:**

In total, 16 776 samples were divided into three groups according to the DD levels detected by Sysmex CS5100 and CA7000: Group A, DD ≥ 2.0 μg/mL fibrinogen equivalent unit (FEU); group B, 0.5 < DD < 2.0 μg/mL FEU; and group C, DD ≤ 0.5 μg/mL FEU. The 95% CIs of the FDP/DD and Fib/DD ratios were calculated. Six abnormal DD results were found according to the 95% CIs. For verification, we performed multiple dilutions, compared the results with those of other instruments, and tested the addition of heterophilic blocking reagent (HBR).

**Results:**

The median and 95% CI of the FDP/DD ratio were 3.76 and 2.25‐8.15 in group A, 5.63 and 2.86‐10.58 in group B, 10.23 and 0.91‐47.71 in groups C, respectively. For the Fib/DD ratio, the 95% CIs was 0.02‐2.21 in group A, 0.68‐8.15 in group B, and 3.82‐55.27 in groups C. Six abnormal results were identified after multiple dilutions, by comparison with other detection systems, and after HBR addition.

**Conclusions:**

The FDP/DD ratio is more reliable for identifying false results. If the FDP/DD ratio falls outside the 95% CI, it should be verified by different methods.

## 1 INTRODUCTION

D‐dimer is a specific degradation product derived from fibrinolytic cross‐linked fibrin clots and mainly reflects fibrinolysis.^1^ The production of D‐dimer requires the sequential activity of three enzymes: thrombin, factor XIIIa, and plasmin.^2^ When blood coagulation occurs, thrombin acts on fibrinogen and transforms into cross‐linked fibrin under the action of factor XIIIa. At the same time, the fibrinolytic system is activated, and plasmin cleaves the substrate fibrin at a specific site, forming D‐dimer.^2^, ^3^ Fibrin degradation product (FDP) is the general term for the degradation products produced after the decomposition of fibrin or fibrinogen under the action of plasmin during hyperfibrinolysis.^4^ FDPs include fibrinogen degradation products (FgDPs) and cross‐linked fibrin degradation products (FbDPs).^5^, ^6^ The former are the products of fibrinogen (Fib) and fibrin monomer (FM), while the latter include the products of D‐dimer and other fragments.^6^ An elevated level of FDP indicates hyperfibrinolysis activity, including primary hyperfibrinolysis and secondary hyperfibrinolysis. Increased D‐dimer formation indicates the presence of thrombosis and secondary hyperfibrinolysis in the body, such as disseminated intravascular coagulation (DIC).^7^ Because D‐dimer is highly sensitive and has a high negative predictive value, its measurement is used to exclude pulmonary embolism (PE), venous thromboembolism (VTE), and deep venous thrombosis (DVT).^8^, ^9^

With the development of modern medicine, many different D‐dimer analysis methods have been developed. Generally, these assays use monoclonal antibodies to detect epitopes that are present in the factor XIIIa‐cross‐linked fragment D domain of fibrin, including methods based on fluorescence, hemagglutination, chemiluminescence, or other techniques.^2^, ^10^, ^11^ The detection methods for D‐dimer in the clinical laboratory mainly include immunoturbidimetry, enzyme immunoassays, and immunochromatography, with the most widely used being immunoturbidimetry. Although immunoassays are commonly used in clinical laboratories, laboratory workers often obtain immunoassay results that are inconsistent with the clinical symptoms. The reason may interference in the immunoassay, including high‐dose hook effects and the presence of heterophilic antibodies, autoantibodies, and cross‐reactive substances.^12^ False‐positive D‐dimer results may lead to misdiagnosis, overtreatment, or missed treatment opportunities for other conditions. Similarly, false‐negative D‐dimer results may result in a missed opportunity for VTE treatment, causing irreversible harm to patients. D‐dimer results that are inconsistent with the clinical symptoms are not uncommon in daily clinical work. Results are sometimes obtained showing normal FDP values but abnormally high D‐dimer levels or low D‐dimer values that are inconsistent with clinical symptoms. We hypothesized that the 95% confidence intervals (CIs) of FDP/DD, Fib/DD ratio can be calculated and used to identify these false results, which will be beneficial to correct these by multiple dilutions.

## 2 MATERIALS AND METHODS

### 2.1 The 95% CIs (confidence intervals) of the FDP/DD ratio and the Fib/DD ratio

A total of 16 776 samples were in rolled in this study. All samples were collected from clinical patients, and the test parameters of all patients included D‐dimer, FDP, and Fib. These patients were in one of the following conditions: pregnancy, hematological disease, postoperative, or other diseases that may have primary/secondary fibrinolysis. D‐dimer, Fib, and FDP measurements performed with Sysmex CS5100 and CA7000 (INNOVANCE^®^ D‐Dimer for D‐dimer, Dade Thrombin Reagent for Fib and Latex Test BL‐2 P‐FDP for FDP). D‐dimer and FDP were detected with latex‐enhanced immunoturbidimetric immunoassays, and Fib was detected with Clauss at the First Affiliated Hospital of Shantou University Medical College from January 2018 to December 2018. The samples were divided into three groups according to D‐dimer level (Table 1). Group A (D‐dimer (DD) ≥2.0μg/mL fibrinogen equivalent unit (FEU)) was composed of 5,186 patients with a median age of 61 years, including 3111 males and 2075 females; group B (0.5 < DD < 2.0μg/mL FEU) was composed of 5037 patients with a median age of 59 years, including 2921 males and 2116 females; and group C (DD ≤ 0.5μg/mL FEU) was composed of 6553 patients with a median age of 52 years, including 3175 males and 3378 females. The 95% CIs of the FDP/DD ratio and the Fib/DD ratio in each group were calculated. The results of the normality test showed that the FDP/DD and Fib/DD ratios were skewed. Therefore, the median and quartile Q (P25, P75) are used to represent the measured data. As increased or decreased FDP/DD and Fib/DD ratios are of clinical significance, the 95% CIs were established using the bilateral percentile method (P2.5‐P97.5) recommended by the Clinical and Laboratory Standards Institute (CLSI).^13^ According to the 95% CIs, some abnormal results were identified, and the samples were then diluted multiple times.

**Table 1:** The D‐dimer level and the 95% CI of the FDP/DD ratio and the Fib/DD ratio in each group

Group	Number of patients	D‐dimer (μg/mL FEU)	FDP/DD ratio [M (P_25_, P_75_)]	Fib/DD ratio [M (P_25_, P_75_)]	FDP/DD ratio 95% CI	Fib/DD ratio 95% CI
Group A	5186 (30.91%)	≥2.0	3.76 (3.22,4.50)^bc^	0.53 (0.23,1.06)^bc^	2.25‐8.15	0.02‐2.21
Group B	5037 (30.03%)	0.5 < DD < 2.0	5.63 (4.65,7.20)^ac^	3.10 (1.99,4.46)^ac^	2.86‐10.58	0.68‐8.15
Group C	6553 (39.06%)	≤0.5	10.23 (6.00,16.47)^ab^	12.43 (8.16,19.71)^ab^	0.91‐47.71	3.82‐55.27

Note

Compared with group A, ^a^ *P* < .05; compared with group B, ^b^ *P* < .05; compared with group C, ^c^ *P* < .05.

Abbreviations: 95% CI, 95% confidence interval; DD, D‐dimer; FDP, fibrin degradation product; FEU, fibrinogen equivalent unit; Fib, fibrinogen.

### 2.2 Abnormal plasma sample results

Patient I was a woman diagnosed with placental abruption, and patients II and III were both male and were diagnosed with thrombocytopenia and leukocytosis. After coagulation testing, D‐dimer levels were found to be slightly increased in the plasma samples of these three patients, while FDP was also significantly increased, and the degree of increase in both parameters were abnormal (Table 2).

**Table 2:** D‐dimer, FDP, and Fib levels and their ratios in patients I, II, and III and patients IV, V, and VI

Patient	Sex	Age (y)	Clinical diagnosis	D‐dimer (μg/mL FEU)		FDP (μg/mL)		Fib (g/L)	FDP/DD ratio		Fib/DD ratio	
				Original	Diluted	Original	Diluted		Original	Diluted	Original	Diluted
I	Female	21	Placental abruption	7.76	264.62	389.40	777.00	0.13	50.18	2.94	0.0168	0.0005
II	Male	23	Thrombocytopenia	2.28	335.73	874.80	1761.10	0.94	383.68	5.25	0.4123	0.0028
III	Male	59	Leukocytosis	9.75	161.37	155.00	432.00	1.32	158.97	2.68	0.1354	0.0082
IV	Female	87	Renal insufficiency	114.23	8.96	12.30	‐	3.83	0.11	1.37	0.0335	0.4275
V	Female	86	Anemic dizziness	336.36	30.40	3.70	‐	2.96	0.01	0.12	0.0088	0.0974
VI	Female	70	Asthma	4.58	0.37	1.50	‐	2.72	0.33	4.05	0.5939	7.3514

Abbreviations: DD, D‐dimer; FDP, fibrin degradation product; FEU, fibrinogen equivalent unit; Fib, fibrinogen.

Patients IV, V, and VI were all elderly women diagnosed with renal insufficiency, anemic dizziness, and asthma, respectively. After the detection of coagulation, D‐dimer was abnormally increased in the plasma samples from these three patients, while the FDP was normal or only slightly increased (Table 2).

#### 2.2.1 Dilution test

The abnormal plasma samples were subjected to serial dilutions (2‐, 4‐, 8‐, 16‐, 32‐, 64‐fold) with INNOVANCE^®^ D‐Dimer DILUENT for D‐dimer, measured by using commercial latex‐enhanced immunotitration (Siemens AG SYSMEX CS‐5100, INNOVANCE^®^ D‐Dimer).

#### 2.2.2 Comparative testing

The plasma samples of patients IV, V, and VI were simultaneously tested with a different instrument using a latex‐enhanced immunoturbidimetric immunoassay (INNOVANCE D‐dimer assay using a SYSMEX CS‐ 5100 and HemosIL D‐dimer HS assay using an ACL TOP700) to determine the D‐dimer value.

#### 2.2.3 Heterophilic antibody blocking reagent (HBR)

The samples of patients IV, V, and VI were treated with a heterophilic blocking reagent (HBR, Scantibodies Laboratory Inc) following the manufacturer's instructions. Briefly, the HBR was immediately thawed in a water bath, gently shaken in an upright position and then added directly to the experimental sample. After incubation at the temperature indicated in the instructions, a D‐dimer assay was performed. The control samples were identical to the test samples except that no HBR was added.

### 2.3 Statistical analysis

Statistical analysis of the data was performed using SPSS 19.0 software (SPSS Inc). The Kolmogorov‐Smirnov test was used to test for the normality of the distributions of the FDP/DD and Fib/DD ratios. The Kruskal‐Wallis *H* test was used to determine the differences between groups, with α = 0.05 as the test level and *P* < .05 indicating differences with statistical significance. GraphPad Prism version 8 (GraphPad Software), Microsoft Word, and Excel were used for creating plots.

## 3 RESULTS

**Figure 1:**
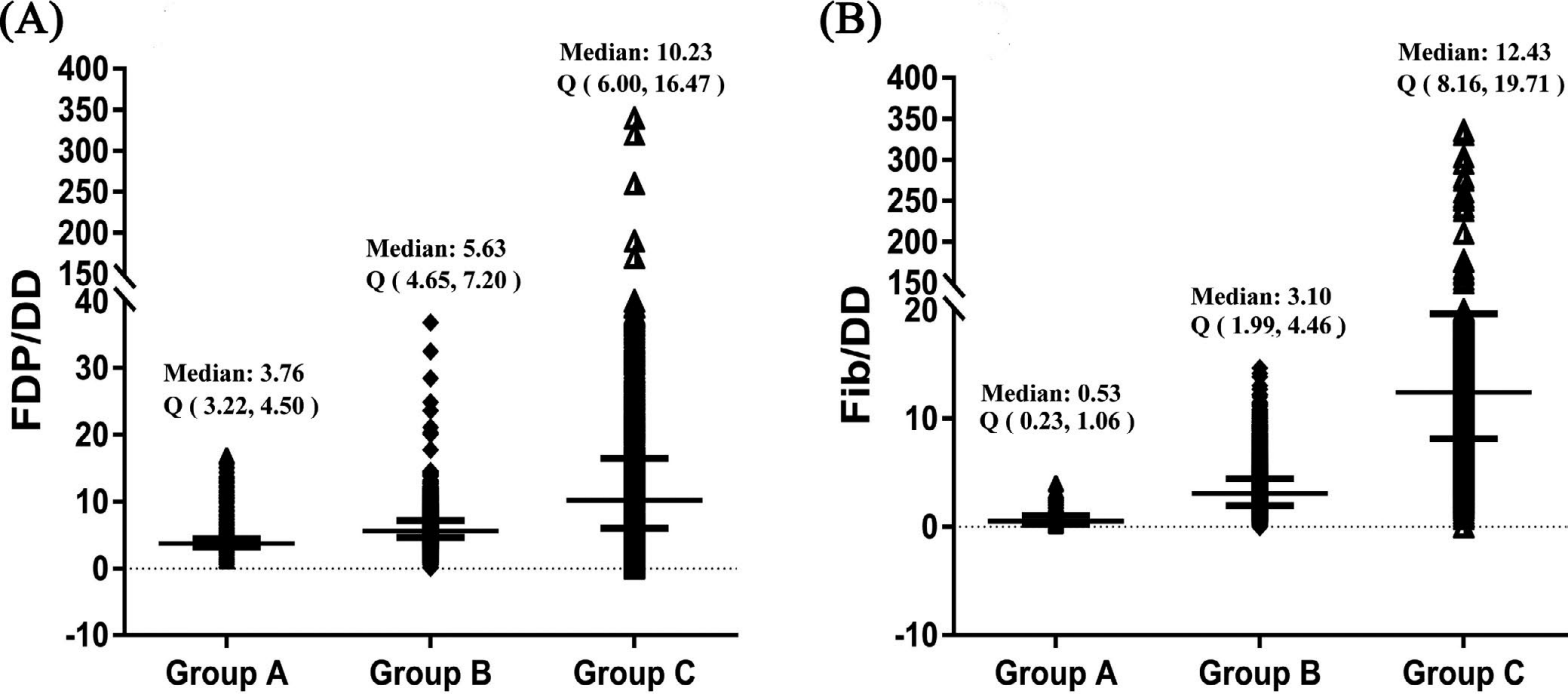
A, Statistical results of the FDP/DD ratio in groups with different D‐dimer levels; B, statistical results of the Fib/DD ratio in groups with different D‐dimer levels. DD, D‐dimer. The *x*‐axis shows the three D‐dimer groups. Group A, DD ≥ 2.0 μg/mL FEU; Group B, 0.5 < DD < 2.0 μg/mL FEU; and Group C, DD ≤ 0.5 μg/mL FEU

Since the FDP/DD ratio and the Fib/DD ratio did not conform to normal distributions, the data are shown as medians and quartiles (P25, P75). Scatter plots of the FDP/DD and Fib/DD ratios and the statistical distributions of the three groups are shown in Figure 1, and the 95% CIs are expressed by the 2.5 and 97.5 quantiles. As seen from the results of group A, the median FDP/DD ratio was 3.76, and the 95% CI was 2.25‐8.15. The median FDP/DD ratio in group B was 5.63, and the 95% CI was 2.86‐10.58. Similarly, the median FDP/DD ratio in group C was 10.23, and its 95% CI was calculated to be 0.91‐47.71 (Figure 1A, Table 1). Figure S1 shows the distribution as a scatter diagram (described in the Supplementary Files). By calculating the Fib/DD ratio, we could also calculate the 95% CI based on the 2.5 and 97.5 quantiles (Figure 1B, Table 1). As shown in Table 1, the 95% CI of the Fib/DD ratio in group A was 0.02‐2.21, while that in group B was 0.68‐8.15. Similarly, the 95% CI for group C was 3.82‐55.27. The scatter diagram for the Fib/DD ratio distribution is shown in Figure S2 (described in the Supplementary Files). Comparing the FDP/DD and Fib/DD ratios among groups with different D‐dimer levels, higher D‐dimer levels were found to correspond to lower FDP/DD and Fib/DD ratios, and the difference was statistically significant (*P* < .05; Table 1).

**Figure 2:**
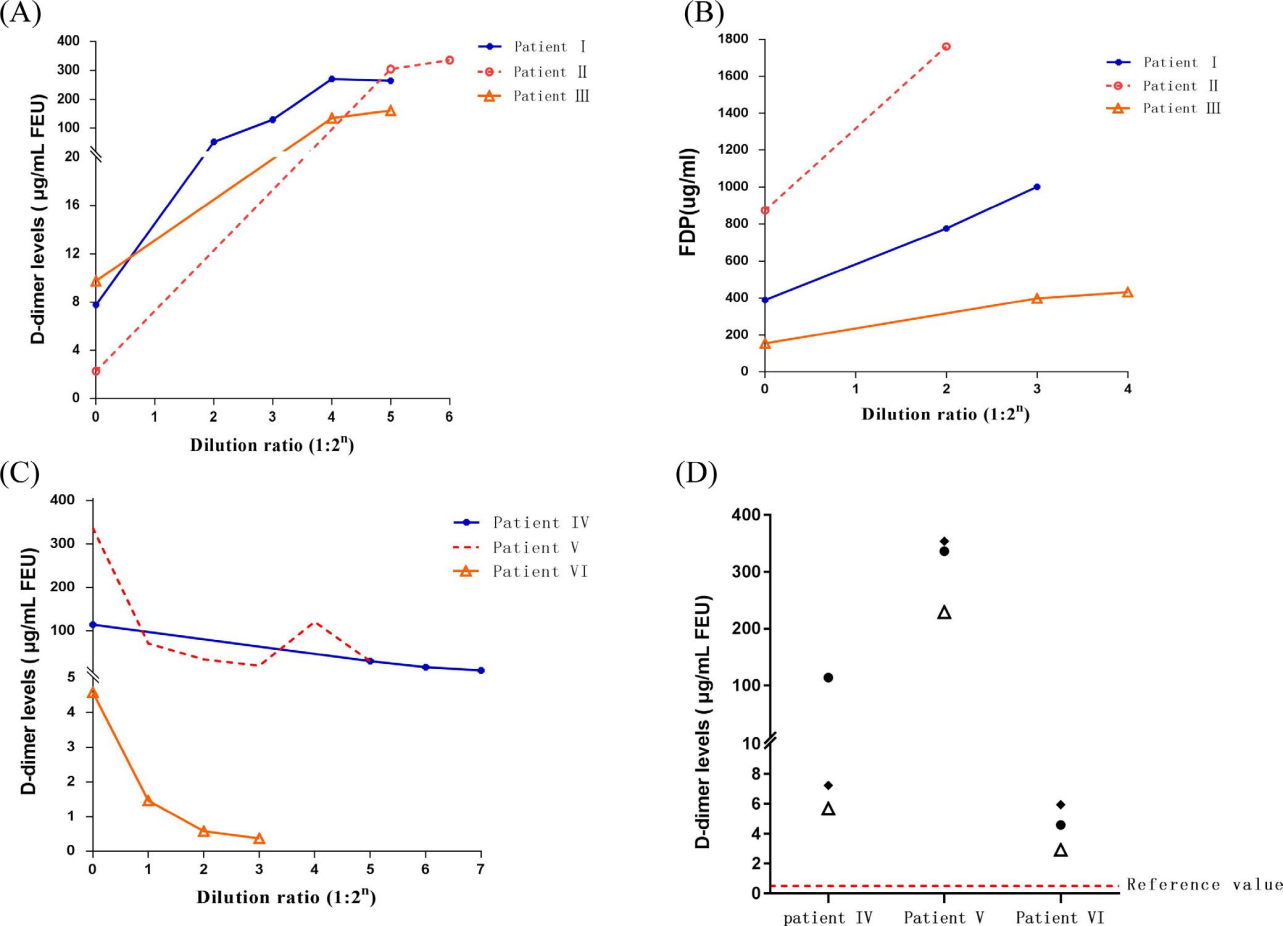
A, Dilution values for patients I to III. After diluting serum samples 4‐, 8‐, 16‐, 32‐, and 64‐fold, the D‐dimer assay was performed, and the results are plotted in the figure. B, Dilution values for patients I to III. After diluting serum samples 4‐, 8‐, 16‐, and 32‐fold, the FDP levels were quantified, and the results are plotted in the figure. C, Dilution values for patients IV to VI. After diluting serum samples 2‐, 4‐, 8‐, 16‐, 32‐, 64‐, and 128‐fold, the D‐dimer assay was performed, and the results are plotted in the figure. D, D‐dimer changes in patients IV to VI. The black circles (●) represent the D‐dimer levels measured in the initial plasma samples; the white triangles (△) represent the levels obtained after treatment with heterophilic blocking reagent (HBR); and the squares (◆) represent the levels in the control group (except for the absence of HBR, the other conditions were the same as the sample group)

In patients I to III, the dilution test showed that the D‐dimer level significantly increased. The D‐dimer level of patient I changed from 7.76 to 264.62 μg/mL FEU after a 32‐fold dilution, whereas that of patient II increased from 2.28 to 335.73 μg/mL FEU after a 64‐fold dilution. Similarly, the D‐dimer level for patient III increased from 9.75 to 161.37 μg/mL FEU after a 32‐fold dilution (Figure 2A, Table 2). Moreover, the FDP values of the three patients also increased after appropriate dilution (Figure 2B, Table 2). In contrast, the initial D‐dimer level of patient IV was as high as 114.23 μg/mL FEU, and the final result was 8.96 μg/mL FEU after being diluted 128‐fold. The D‐dimer result of patient V decreased from 336.36 to 30.40 μg/mL FEU after a 32‐fold dilution and that of patient VI decreased from 4.58 to 0.37 μg/mL FEU after 8‐fold dilution (Figure 2C, Table 2). The Fib/DD ratios are similar, as shown in the Table 2. For specimens with increased pseudomorphic D‐dimer levels, we used different instruments to perform comparative experiments. The plasma samples of patients IV to VI measured by latex‐enhanced immunoturbidimetric immunoassay were within the normal reference range (Table 3). We also detected D‐dimer levels after adding HBR to eliminate interference. The results showed that the D‐dimer levels of the three samples were significantly decreased after HBR addition (Figure 2D).

**Table 3:** Comparative D‐dimer test

Patient	Test					Comparative test				
	D‐dimer (μg/mL FEU)			FDP (μg/mL)		D‐dimer (μg/mL FEU)			FDP (μg/mL)	
	Test methodology	Value	Reference range	Value	Reference range	Test methodology	Value	Reference range	Value	Reference range
IV	INNOVANCE^®^ D‐Dimer, latex‐enhanced immunoturbidimetric immunoassay	114.23	<0.50	12.30	0‐5	HemosIL D‐dimer, latex‐enhanced immunoturbidimetric immunoassay	3.79	<0.50	11.50	0‐5
V		336.36		3.70			0.49		1.10	
VI		4.58		1.50			0.15		0.70	

Abbreviations: DD, D‐dimer; FDP, fibrin degradation product; FEU, fibrinogen equivalent unit.

## 4 DISCUSSION

As a specific degradation product of cross‐linked fibrin, D‐dimer is a specific indicator of thrombosis and secondary fibrinolysis. If the determined D‐dimer level exceeds the recommended cutoff value of the kit, it cannot be used as the only criterion for the diagnosis of acute PE, DVT, and DIC; rather, it must be comprehensively analyzed in combination with the clinical conditions.^14^ The reason is that as long as there is activated thrombosis, namely, active fibrinolysis, in the blood vessels of the body, the D‐dimer levels will be increased. For example, surgery, tumor, infection, and tissue necrosis can lead to an increase in the D‐dimer level. As a noninvasive examination method, the D‐dimer level is important for the exclusion of PE, DVT, and DIC diagnoses.^15^ D‐dimer detection has played an important role in clinical practice.^14^ False‐positive or false‐negative D‐dimer results may lead to misdiagnosis, overtreatment for related diseases, or missed opportunities for appropriate treatment.

Because FDP is the general term for fibrinogen and fibrin degradation products, it includes D‐dimer; thus, the combination of D‐dimer and FDP can eliminate some of the false D‐dimer results.^4^ Recent studies indicate that DD, Fib, and their ratios with FDP can also be used for disease prediction. The FDP/Fib and DD/Fib ratios can be used to predict high‐risk patients with fatal thrombosis complications of acute abnormal promyelocytic leukemia (APL) and to predict the presence of pelvic fractures caused by arterial extravasation.^16^, ^17^ Changes in the FDP/Fib and DD/Fib ratios are more significantly correlated with Fib, FDP, or DD levels and may be helpful in designing lower risk treatment regimens or personalized therapies.^10^, ^18^, ^19^ Therefore, in this study, we calculated the 95% CIs of different D‐dimer results and used large‐scale data to calculate the FDP/DD ratio. The statistical results show that when the D‐dimer level is higher than 0.5 μg/mL FEU, the FDP/DD values are lower, and the 95% CI range will be smaller, which can better reflect the actual concentration. When the FDP/DD ratio exceeds this range, attention should be paid to whether there are false results for the D‐dimer measurements, and we must verify that the results are accurate. In addition, when the D‐dimer level is less than 0.5 μg/mL FEU, the variation in the FDP/DD ratio is larger, as shown in the scatter plot by the more scattered distribution of the data. This result occurs because when the D‐dimer level is lower than the cutoff value, the relative effect of the denominator on the value is greater. If there are no corresponding clinical symptoms, hyperfibrinolysis can be directly ruled out in the normal ranges of D‐dimer and FDP, and thus, the FDP/DD ratio is not important. In addition, because FDP is not measured in some patients at the time of D‐dimer testing, when we encountered plasma samples suspected of producing false D‐dimer results, we could not preliminarily judge whether the results were reliable according to the FDP/DD ratio. Therefore, we also calculated the Fib/DD ratio to determine whether this ratio could be used instead of the FDP/DD ratio as a reference. However, we found that for some abnormal results, the Fib/DD ratio did not exceed the 95% CI range, indicating that the Fib/DD ratio cannot be used. Therefore, to identify false D‐dimer results as early as possible, one needs to detect D‐dimer and FDP together.

During the statistical analysis, we noticed that most of the abnormal results came from the ICU and the clinical departments of hematology, rheumatism, neurology, and infectious diseases. Therefore, we selected several samples for specific analysis to determine whether the results were reliable and how to identify false‐positive or false‐negative results. In patients I to III, the initial D‐dimer levels were significantly lower than the FDP results, and the results after dilution were increased by ten‐fold or more. In these patients, the D‐dimer test produced false‐negative results. Similar cases have been reported in the relevant literature.^20^ Because the principle of D‐dimer detection relies on antibody recognition, false negatives easily arise because of inappropriate proportions of antigens and antibodies, termed the hook effect, which is mainly due to antigen overabundance. A solution to this problem is to dilute the plasma sample and retest at multiple dilutions. As shown in Table 2, the D‐dimer results of the plasma samples from patients I to III increased dramatically after dilution, and the FDP/DD ratio also dropped to within the normal range. Considering the D‐dimer results of patients IV, V, and VI, the original D‐dimer levels were significantly increased, while the FDP values were normal or slightly increased. However, when we diluted the samples, the D‐dimer results were found to significantly decrease, even to within the normal reference range, and the FDP/DD ratio calculated after dilution was within a reasonable range. However, there was no significant linear relationship between the D‐dimer level and the dilution ratio, indicating that the original D‐dimer results were unreliable.^19^ Therefore, the D‐dimer results of these samples were false positives. The cause of the false‐positive results can be revealed from the experimental results, which show that the same plasma tested by different instruments and methods can yield significantly different D‐dimer measurement results (Table 3). However, the cause of this discrepancy remains unclear.^21^ Under normal circumstances, there are many factors that could lead to abnormal D‐dimer results, including abnormal specimens, poor instrument quality control, and the presence of heterophilic antibodies; previous articles have also reported that the presence of rheumatoid factors can affect D‐dimer results.^22^ Interference with heterophilic antibodies is the most common factor after instrument and specimen problems are eliminated.^19^, ^23^, ^24^, ^25^, ^26^ Our study revealed that after eliminating the effects of heterophilic antibodies with HBR, the plasma sample results of patients IV to VI remained higher than the reference range but significantly decreased, while there was no significant change in the control group. This finding suggests that the abnormal increase in D‐dimer results in these patients does indicate some form of interference; heterophilic antibodies have a significant effect on this interference, while at the same time, there may be other influencing factors.

In this study, the D‐dimer, FDP, and Fib levels of 16 776 patients from January 1, 2018, to November 30, 2018, were retrospectively analyzed and compared; we preliminarily established the 95% CIs for the FDP/DD and Fib/DD ratios. As this study is based on a large sample size, it has certain credibility, which is also a highlight of this paper. By monitoring the FDP/DD and Fib/DD ratios, researchers and clinicians can determine whether the ratios are within the 95% CIs or whether the ratios are inverted, allowing them to intuitively judge the reliability of the results and whether the elimination of additional sources of interference is needed.

Taken together, when significant abnormalities in the FDP/DD ratio are encountered, the results should be verified to eliminate sources of unnecessary interference with clinical symptoms. If necessary, some corresponding problems can be solved by eliminating interference by heterophilic antibodies. We also suggest that each laboratory establishes its own reference ranges for the FDP/DD ratio so that it could be used to monitor whether the D‐dimer level is a false result. However, when laboratory conditions are limited regarding the determination of false‐positive or false‐negative results, the simplest and most straightforward solution is to serially dilute the samples.

## CONFLICT OF INTEREST

No conflict of interest exists in the submission of this manuscript, and the manuscript has been approved by all authors for publication.

## AUTHORS' CONTRIBUTIONS

Xianyan Zhang and Xuexuan Zhang participated in writing the article and performing the statistical analysis of the experimental data. Jialong Xu and Tengyi Huang are mainly responsible for data collection. Ying Wu was responsible for the heterophilic antibody experiment. Yeru Yang and Huanbin Zhou were responsible for the dilution test and the comparative test. Yinge Wu was responsible for the design of the experiment and the final assembly of the article.
