# Supplementary figures and images for: Identification of and solution for false D‐dimer results
Source: J Clin Lab Anal. 2020 Jan 22;34(6):e23216. doi: 10.1002/jcla.23216 (PMC7307351; doi:10.1002/jcla.23216)

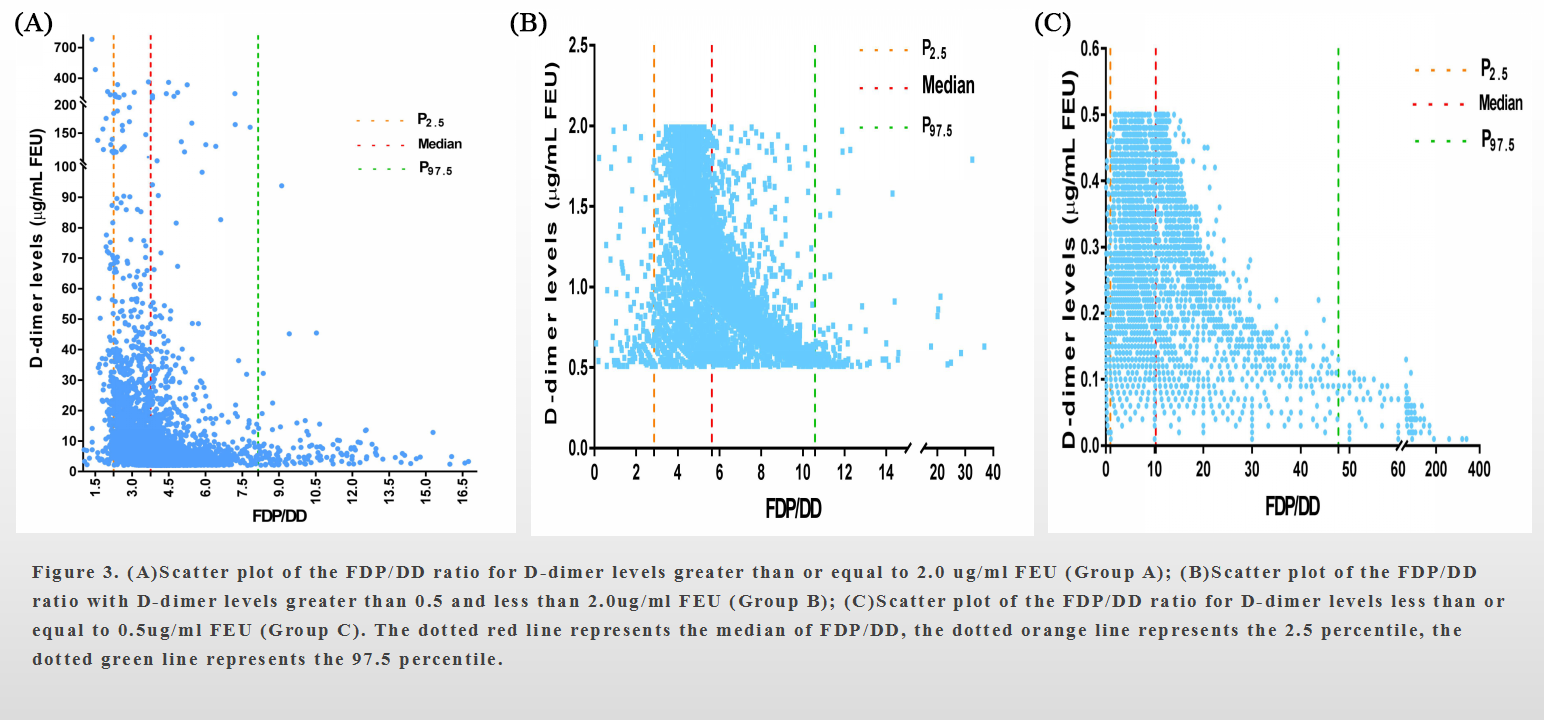

Supplement: Supplementary file 1 [file JCLA-34-e23216-s001.tiff]

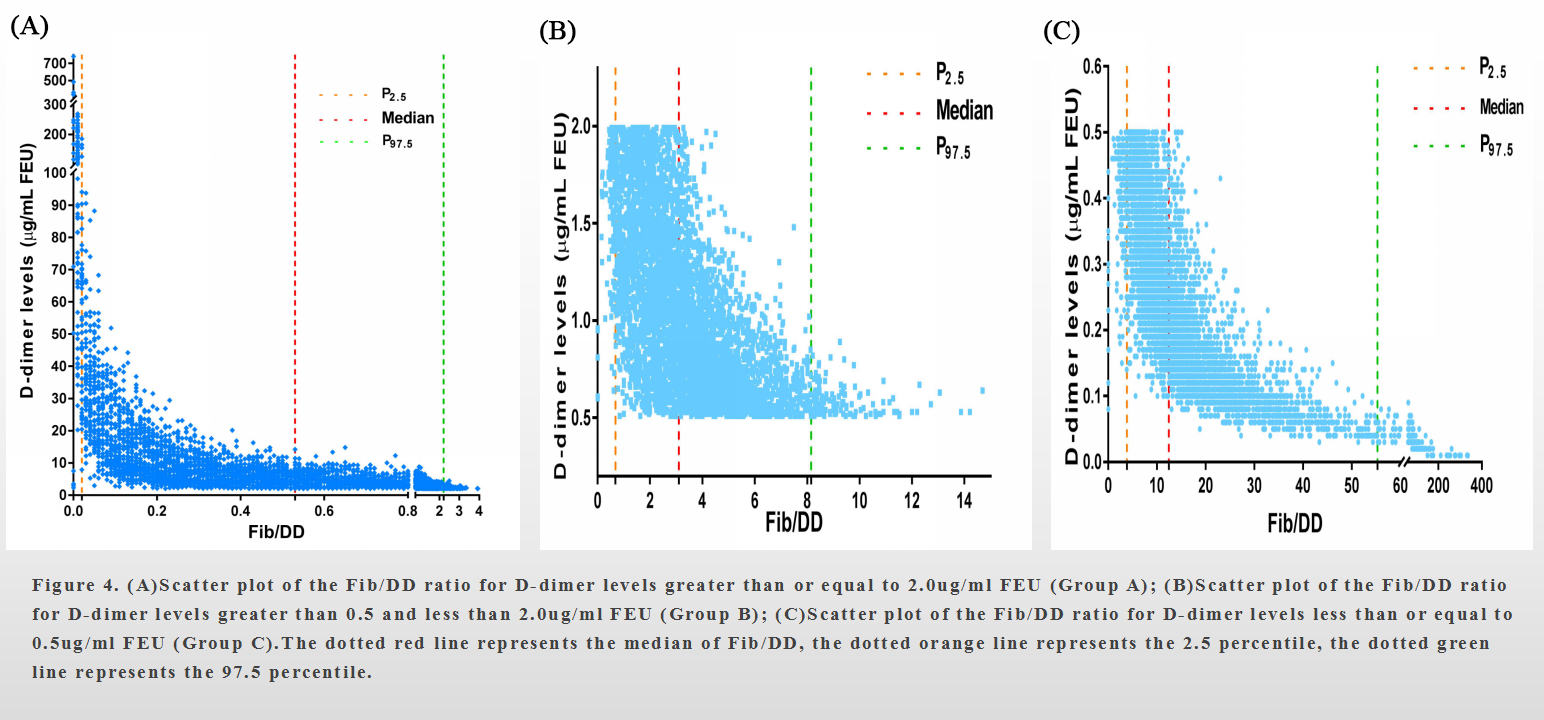

Supplement: Supplementary file 2 [file JCLA-34-e23216-s002.tiff]
